# Scandcleft randomized trials of primary surgery for unilateral cleft lip and palate: comparison of dental arch relationships and dental indices at 5, 8, and 10 years

**DOI:** 10.1093/ejo/cjab055

**Published:** 2021-09-03

**Authors:** Arja Heliövaara, Annelise Küseler, Pål Skaare, Haydn Bellardie, Kirsten Mølsted, Agneta Karsten, Agneta Marcusson, Sara Rizell, Eli Brinck, Paul Sæle, Midia Najar Chalien, Jeanette Mooney, Phil Eyres, William Shaw, Gunvor Semb

**Affiliations:** Cleft Palate and Craniofacial Center, Department of Plastic Surgery, Helsinki University Hospital and Helsinki University, Finland; Cleft Palate Center and University Hospital Aarhus and University of Aarhus, Denmark; Department of Plastic and Reconstructive Surgery, Oslo University Hospital Rikshospitalet, Norway; Dental School, University of Manchester, UK; University of the Western and Cape, Cape Town, South Africa; Copenhagen Cleft Palate Center, University Hospital of Copenhagen, Denmark; Section for Orthodontics, Division of Orthodontics and Pedodontics, Department of Dental Medicine, Karolinska Institutet and Stockholm Craniofacial Team, Sweden; Maxillofacial Unit and Department of Clinical and Experimental Medicine, Linköping University, Sweden; Specialist Clinic for Orthodontics, University Clinics of Odontology, Public Dental Health Service, Västra Götaland Region, Sweden; Department of Plastic and Reconstructive Surgery, Oslo University Hospital Rikshospitalet, Norway; Oral Health Center of Expertise/Western Norway, Bergen, Norway; Specialist Clinic for Orthodontics, University Clinics of Odontology, Public Dental Health Service, Västra Götaland Region, Sweden; Dental School, University of Manchester, UK; Dental School, University of Manchester, UK; Dental School, University of Manchester, UK; Department of Plastic and Reconstructive Surgery, Oslo University Hospital Rikshospitalet, Norway; Dental School, University of Manchester, UK

## Abstract

**Background and trial design:**

The Scandcleft intercentre study evaluates the outcomes of four surgical protocols (common method Arm A, and methods B, C, and D) for treatment of children with unilateral cleft lip and palate (UCLP) in a set of three randomized trials of primary surgery (Trials 1, 2, and 3).

**Objectives:**

To evaluate and compare dental arch relationships of 5-, 8-, and 10-year-old children with UCLP after four different protocols of primary surgery and to compare three dental indices. The results are secondary outcomes of the overall trial.

**Methods:**

Study models taken at the ages of 5 (*n* = 418), 8 (*n* = 411), and 10 years (*n* = 410) were analysed by a blinded panel of orthodontists using the Eurocran index, the 5-year-olds’ (5YO) index, and the GOSLON Yardstick. Student’s *t*-test, Pearson’s correlation, chi-square test, and kappa statistics were used in statistical analyses.

**Results:**

The reliability of the dental indices varied between moderate and very good, and those of the Eurocran palatal index varied between fair and very good. Significant correlations existed between the dental indices at all ages. No differences were found in the mean 5-, 8-, and 10-year index scores or their distributions within surgical trials. Comparisons between trials detected significantly better mean index scores in Trial 2 Arm C (at all ages) and in Trial 1 Arm B (at 5 and 10 years of age) than in Trial 3 Arm D. The mean Eurocran dental index scores of the total material at 5, 8, and 10 years of age were 2.50, 2.60, and 2.26, and those of the 5YO index and GOSLON Yardstick were 2.77, 2.90, and 2.54, respectively. At age 10 years, 75.8% of the patients had had orthodontic treatment.

**Conclusions:**

The results of these three trials do not provide evidence that one surgical method is superior to the others. The reliabilities of the dental indices were acceptable, and significant correlations existed between the indices at all ages. The reliability of the Eurocran palatal index was questionable.

**Trial registration:**

ISRCTN29932826.

## Introduction

Controversy about primary cleft surgery has led to several variations in type, technique, and sequencing of lip and palate surgery in unilateral cleft lip and palate (UCLP). The aim of the Scandcleft intercentre study is to test the outcomes of four surgical protocols for treatment of children with complete UCLP. The project consists of three concurrent randomized trials of primary palatal surgery for infants born with UCLP. The study was developed and executed by ten North European cleft teams: Aarhus/Copenhagen (Denmark), Helsinki (Finland), Bergen/Oslo (Norway), Gothenburg/Linköping/Stockholm (Sweden), Manchester/Belfast (UK). Belfast withdrew at the beginning of the study.

The principal outcome measures of the Scandcleft study are speech and dentofacial development. The hypothesis of the study is that variations in surgical methods, timing, and staging are not associated with difference in outcome in UCLP. The design, background, surgical methods, and comparisons of dental arch relationships with 5-year-olds’ (5YO) index, GOSLON Yardstick and Modified Huddart Bodenham (MHB) index at 5 and 8 years of age have been published earlier (1–6). This paper is a continuation of the study at 10 years of age, and the results are secondary outcomes of the overall trial.

One of the challenges in orthodontic treatment and research of children with UCLP, in early adulthood, is the time lapse between primary surgery and dentofacial outcome. Regardless of the surgical method for primary palatal closure, facial growth in UCLP is characterized by a progressive retrusion of the profile relative to the cranial base involving the nasal bone, the mandible, and especially the maxilla (7, 8). Anterior and lateral crossbites are often present in the deciduous dentition.

Several dental indices have been developed for comparison of surgical methods, auditing the results of primary cleft surgery, and prediction of outcome. The most commonly used index for evaluation of occlusal outcome and severity of crossbite in children with UCLP is the GOSLON Yardstick (9). Other commonly used indices are the 5YO index (10, 11), Eurocran index (12), the Huddart/Bodenham (HB) index (13), and the MHB index (14). The GOSLON Yardstick and the 5YO index categorize the occlusal outcome into one of five categories from excellent to very poor. They assess anteroposterior, vertical, and transversal relationships, with the first being most important. The GOSLON Yardstick has been developed to grade dental arch relationships in the late mixed/early permanent dentition. The 5YO index is applied in the deciduous dentition.

The Eurocran index is a modification of the GOSLON Yardstick and 5YO index (12). It assesses dental arch relationships with a 4-point scale and in addition, it evaluates palatal morphology with a 3-point scale. The scar tissue that develops over the denuded bone after palatoplasty has been assumed to contribute to the growth disturbance (15). The Eurocran index is the only index that assesses two components: the occlusal relationship in all three planes of space (including displacement of the lesser segment on the cleft side) and the palatal morphology (16). Variants of this index have been developed for application in either the 5- or 9-year age group (12).

Whereas the 5YO, Eurocran indices and the GOSLON Yardstick were developed for categorizing the degree of malocclusion, the HB and MHB indices use linear scales to measure maxillary arch constriction. The MHB index scores each maxillary tooth and its opposing tooth based on presence and degree of crossbite. These scores are summed to produce the overall score. The more negative the score, the more severe the crossbite.

The 5YO, Eurocran indices, and the GOSLON Yardstick have proven to be reliable and capable of discriminating the quality of dental arch relationships in children with ULCP within and between centres and treatment protocols (3, 4, 9–12, 17–20). However, the most comprehensive outcome measure (21) and how the results differ between measures remain mostly unknown. In addition to the evaluation of surgical methods, the large patient material of the Scandcleft intercentre study provides an opportunity for assessment and comparison of dental indices.

The purpose of this Scandcleft paper was to evaluate the occlusal outcome of UCLP after four different protocols of primary surgery at 5, 8, and 10 years of age. The additional aim of the research was to compare the Eurocran index with the 5YO index and the GOSLON Yardstick. The hypotheses were that variations in surgical method, timing, and staging are not associated with different outcomes in UCLP, and the results do not differ between the different indices.

## Materials and methods

**Figure 1.**
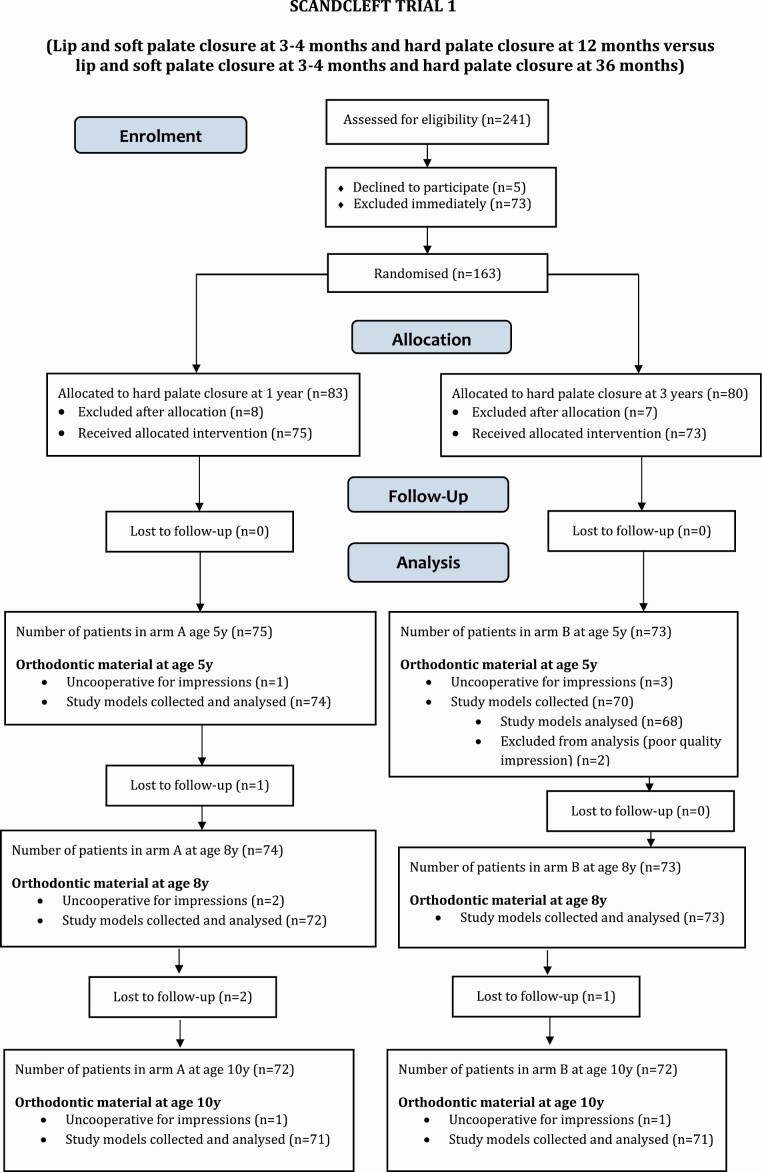

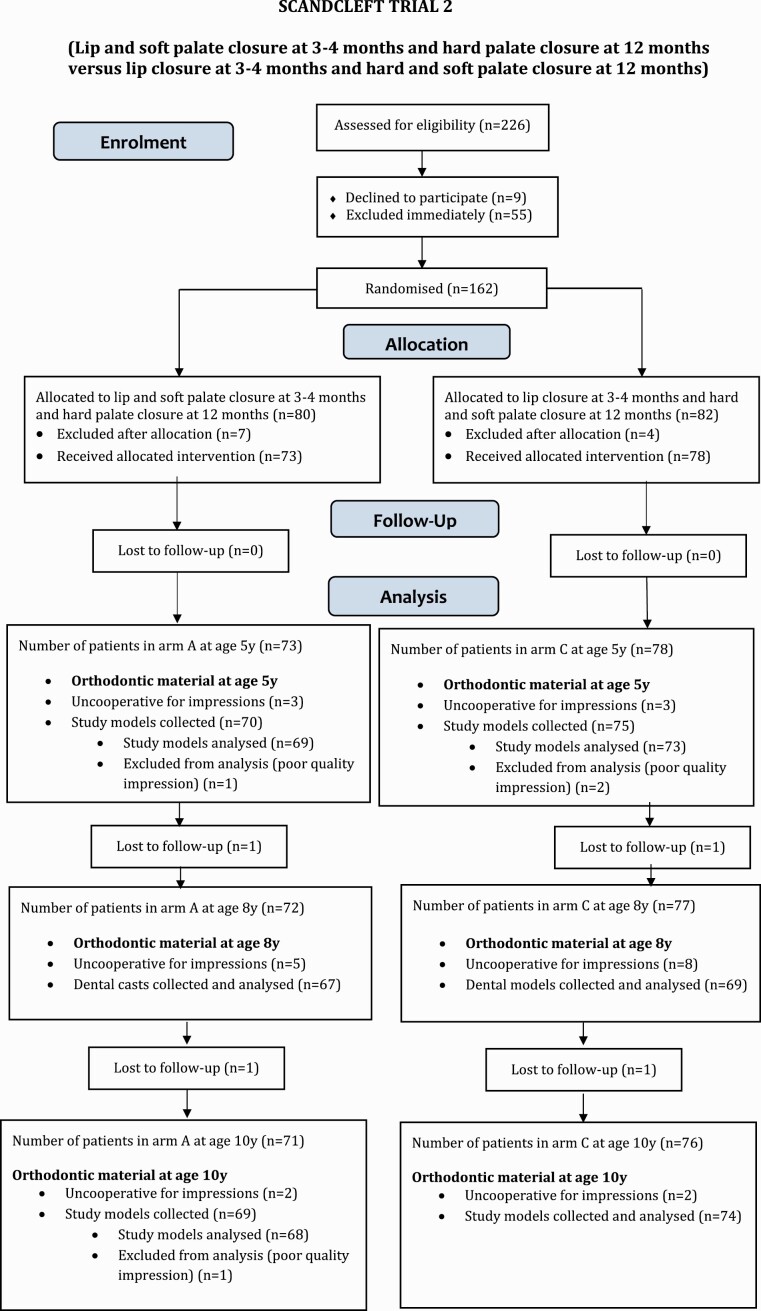

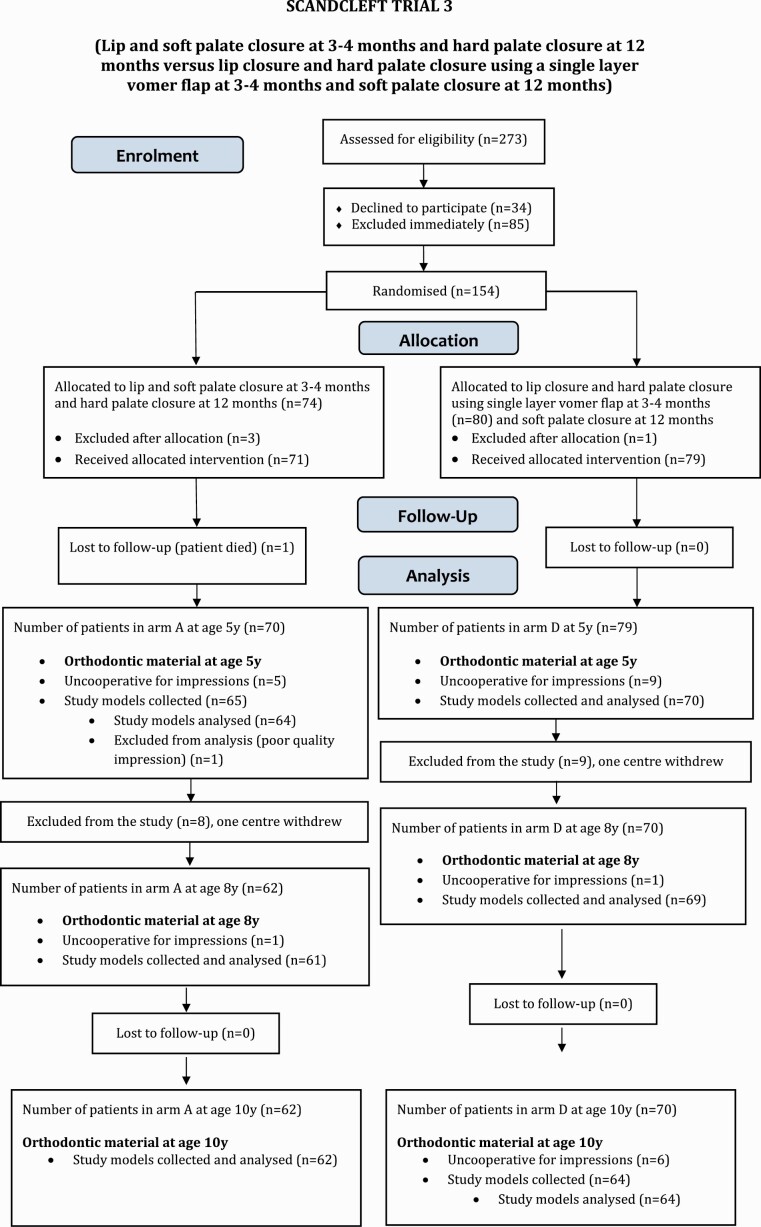
Flow charts of the three Scandcleft trials.

**Figure 2.**
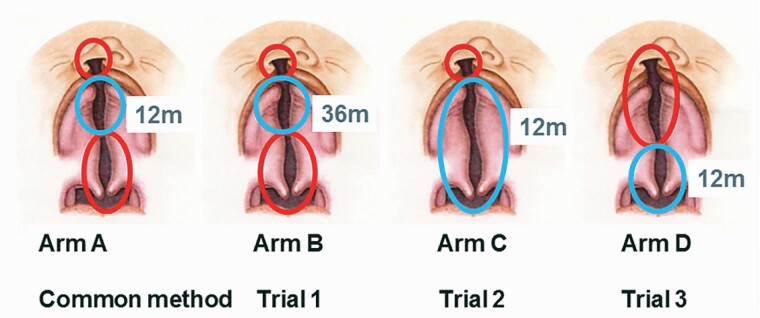
Sequence of closure of unilateral cleft lip and palate (UCLP). The figure and text are reprinted with permission from Rautio *et al.* (2017), *Journal of Plastic Surgery and Hand Surgery*, Taylor & Francis. The first operation is indicated by the red line and the second operation by the blue line. In the common method (Arm A), the lip was repaired simultaneously with soft palate closure at 3–4 months, followed by hard palate closure at 12 months. In Trial 1, the common method (Arm A) was compared with hard palate closure delayed until 3 years of age (Arm B). In Trial 2, the common method (Arm A) was compared with closure of the lip at 3–4 months, followed by closure of the hard and soft palate together at 12 months (Arm C). In Trial 3, the common method (Arm A) was compared with lip and hard palate closure at 3–4 months, followed by soft palate closure at 12 months (Arm D).

Nine cleft centres in Denmark, Finland, Norway, Sweden, and the UK participated in a set of three randomized trials of primary surgery. Three groups of centres (Trials 1, 2, and 3) tested surgical methods (Arms B, C, and D) against a common method (Arm A). The background of the study, material (1), and surgical methods (2) are described in more detail in separate papers. The flow charts of the three Scandcleft trials are presented in Figure 1. A description of the sequence of surgical closure is given in Figure 2. After the primary operations of the cleft, the patients attended regular follow-up evaluations of the cleft team at the cleft centres until early adulthood. In this paper the dental casts and the information of the 5-, 8-, and 10-year follow-ups are used.

The number, gender, and age of patients at ages 5, 8, and 10 years are presented in Table 1. Originally, 448 Caucasian patients attended the 5-year follow-ups of the Scandcleft study, but 24 children (5.4%) did not have impressions taken because of lack of co-operation. From the 424 models collected, 418 (98.6%) were rated, with six models being excluded from analysis because of poor-quality impressions. At 8 years of age, 429 patients attended the follow-ups of the Scandcleft study. Due to lack of co-operation, 411 patients were analysed, with 18 children (4.2%) not having impressions. At 10 years of age, 410 dental models were analysed, with 10 children (2.4%) not having impressions due to lack of co-operation. One model was excluded from the analysis because of poor-quality.

**Table 1.** Number, gender, and age (years) of patients in the three trials with four surgical methods (Arms A, B, C, and D) at 5, 8, and 10 years of age.

	Trial	Children (boys/girls)	Mean age (range)	Arm A	Arms B or C or D
Age 5 years	Trial 1 (A, B)	142 (96/46)	5.1 (4.8–7.0)	74	68
	Trial 2 (A, C)	142 (94/48)	5.1 (4.8–6.6)	69	73
	Trial 3 (A, D)	134 (83/51)	5.3 (4.9–6.9)	64	70
	Total	418 (273/145)	5.1 (4.8–7.0)	207	211
Age 8 years	Trial 1 (A, B)	145 (99/46)	8.1 (7.5–9.2)	72	73
	Trial 2 (A, C)	136 (89/47)	8.1 (7.4–10.0)	67	69
	Trial 3 (A, D)	130 (82/48)	8.2 (7.0–9.3)	61	69
	Total	411 (270/141)	8.1 (7.0–10.0)	200	211
Age 10 years	Trial 1 (A, B)	142 (96/46)	10.1 (9.6–11.2)	71	71
	Trial 2 (A, C)	142 (94/48)	10.1 (9.2–11.1)	68	74
	Trial 3 (A, D)	126 (77/49)	10.2 (9.9–11.5)	62	64
	Total	410 (267/143)	10.1 (9.2–11.5)	201	209

### Orthodontic treatment

At the age of 5 and 8 years, none of the children had had bone grafting of the alveolar cleft or had had orthodontic treatment. At the age of 10 years, 211 of 410 children (75.8%) had undergone orthodontic treatment [134 of 142 (94.4%) in Trial 1, 100 of 142 (70.4%) in Trial 2, and 77 of 126 (61.1%) in Trial 3]. No presurgical orthopaedics was used. In Trial 1, seven of the children had used nasal elevators and eleven children used nasal plugs in early childhood.

The goals of the bone grafting of the alveolar cleft in the mixed dentition are to separate the oral and nasal cavities, to stabilize and consolidate the maxilla with a bony union, to create adequate bone for region of the upper incisors and canines, and to support the alar base and nose. The orthodontic protocols of the different cleft centres after 8 years of age prior to alveolar bone grafting operation were rather similar. After multidisciplinary team assessment with the surgeons, and agreement to perform bone grafting, presurgical orthodontics was started in the upper arch with a removable appliance or quad helix and / or labial fixed appliances. The orthodontic treatment was planned individually. The goals of the orthodontic treatment included correction of crossbites and transverse and sagittal relationships; alignment, rotation and proclination of upper incisors to establish maxillary arch form and to create room for alveolar bone graft placement. In addition, the lesser dentoalveolar segment was usually rotated laterally as in UCLP the lesser segment is often mesially and distally displaced.

In very poor cases with bilateral crossbite and with reverse overjet with proclined incisors, the crossbite was not corrected at this age as the later orthognathic surgical treatment was planned. Only one small centre in Trial 1 used Delaire face masks prior bone graft for protraction, if needed. Most of the orthodontic treatment was performed in the cleft centres. In one large centre in Trial 2, most of the orthodontic treatment was performed locally according to the instructions of the cleft centre.

### Ratings of the dental models

**Figure 3.**
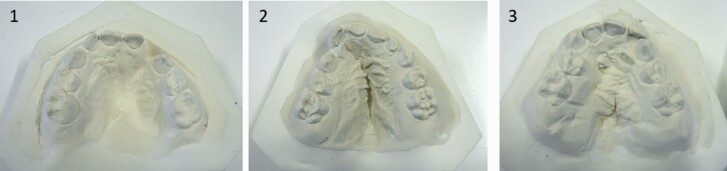
Photographs of examples of the models used for reference of palatal morphology of the Eurocran index scores from grade 1 to 3. Palatal morphology according to the Eurocran index (12). 1. Good anterior and posterior height; minor surface irregularities (bumps and crevices); no or minor deviation of arch form. 2. Moderate anterior and posterior height; moderate surface irregularities (bumps and crevices); moderate deviation of arch form (e.g. segmental displacement). 3. Severe reduction in palate height; severe surface irregularities (bumps and crevices); severe deviation in arch form (e.g. ‘hourglass’ constriction). The worst feature of the three suggests the initial score. This may be modified up or down depending on how good the other features are. If good arch form was achieved by means of orthodontic treatment, the case is graded lower.

Ratings of duplicate dental models using the 5YO and Eurocran indices and the GOSLON Yardstick were done by cleft orthodontists of the participating cleft centres. The ratings were performed in three meetings during years 2012 (rating of the 5-year models), 2015 (rating of the 8-year models), and 2018 (rating of the 10-year models). The ratings were performed during several days by blinded panels of orthodontists who scored independently all models. Customized scoring sheets were used. During the ratings reference models of both palatal morphology (Figure 3) and dental arch relationships of the indices were available to help the raters confirm the appropriate grade in borderline cases. At all ages, 30 randomly selected dental arch relationships and palatal casts were scored twice by all examiners in order to calculate reliability. At age 5 years, ratings with the Eurocran and 5YO indices to assess both dental arch relationships and palatal morphology were performed by a blinded panel of 16 orthodontists who scored all models. At 8 years of age, 11 raters assessed the dental arch relationships with GOSLON Yardstick and seven raters with the Eurocran index. At the age of 10 years, 12 raters assessed the dental arch relationships with the Eurocran index and GOSLON Yardstick as above. Ten same raters assessed all models with the 5YO index and GOSLON Yardstick at 5, 8, and 10 years of age, and 5 same raters with the Eurocran index at all ages. The results of the 5YO index at 5 years and the GOSLON Yardstick at 8 years have been published earlier (3, 4).

### Ethical approval

The research protocol was approved by all centres and local ethical approvals were obtained: Denmark (Aarhus and Copenhagen), Regional Ethics Committee 1997/4121, Finland (Helsinki) 4/9/97, Norway (Bergen and Oslo) S-97152, Sweden (Gothenburg) R257-97, (Stockholm and Linköping) 97-372, UK (Manchester) 99/197 (CM/96/197). Principles outlined in the Declaration of Helsinki were followed.

### Statistical methods

Kappa statistics were calculated to assess reliability within and between examiners. *T*-tests were used in comparisons of the trials. Pearson’s correlation was used to compare the indices with each other. Chi-square statistics was calculated to compare orthodontic treatment between trials. Test statistics with *P*-values equal to or less than 0.05 were considered significant. Kappa values above 0.81 were regarded as very good, values between 0.61 and 0.80 as good, values between 0.41 and 0.60 as moderate, and values below 0.40 as poor (22).

## Results

### Reliabilities of the indices

At age 5 years, good to very good levels of agreement within and between judges were obtained in the Eurocran index and 5YO index for dental relationships (Table 2). The intra-rater mean Eurocran palatal scores varied between 0.30 and 1.00 (fair to very good), and the inter-rater reliability between 0.26 and 0.56 (fair to moderate).

**Table 2.** Index comparisons of the intra- and inter-rater reliabilities at 5, 8, and 10 years of age.

	Intra-rater	Inter-rater
Age 5 years		
Eurocran dental arch relationships	0.71–0.97	0.72–0.92
Eurocran palatal index	0.30–1.00	0.26–0.56
5-Year-olds’ (5Y0) index	0.71–0.94	0.70–0.87
Age 8 years		
Eurocran dental arch relationships	0.81–0.95	0.72–0.80
GOSLON Yardstick	0.62–0.89	0.60–0.80
Age 10 years		
Eurocran dental arch relationships	0.71–1.0	0.52–0.85
GOSLON Yardstick	0.57–0.89	0.43–0.71

At 8 years of age, the intra-rater and inter-rater reliabilities of the Eurocran index varied between good and very good, and those of the GOSLON Yardstick between moderate and good. At 10 years of age, the intra-rater reliability of the Eurocran scores varied between good and very good and the inter-rater reliability between moderate and good. The intra-rater reliability of the GOSLON Yardstick varied between moderate and very good and the inter-rater reliability between moderate and good (Table 2).

### Correlations between dental indices

Significant correlations existed between the Eurocran dental index scores and the 5YO scores at 5 years of age (Pearson correlation 0.960, *P* < 0.01), and the Eurocran and the GOSLON Yardstick scores at 8 and 10 years of age (Pearson correlation 0.951, *P* < 0.01, and Pearson correlation 0.966, *P* < 0.01, respectively).

### Mean index scores and comparison of surgical trials

#### Mean dental index scores

The mean Eurocran dental index scores of the total material at 5, 8, and 10 years of age were 2.50, 2.60, and 2.26, and those of the 5YO index and GOSLON Yardstick were 2.77, 2.90, and 2.54, respectively. No significant differences emerged between boys and girls at any age with any of the indices.

#### Mean Eurocran palatal index score

The mean palatal score of the total material was 1.70 (SD 0.39). The palatal scores varied from 1.62 (Arm D) to 1.73 (Arm A). As the reliability of the palatal index was questionable, no further statistical comparisons were performed.

#### Comparisons within trials

Comparisons within each trial showed no significant differences in the mean 5-, 8-, and 10-year index scores or their distributions between the common method A and methods B, C, and D. A trend of slightly more unfavourable scores with increasing age from 5 to 8 years was noted with all indices (Table 3). At the age of 10 years, 75.8% of the children had had orthodontics and the scores of both the Eurocran index and the GOSLON Yardstick had improved. There was a significant difference in orthodontic treatment between the trials at age 10 years (chi-square 48.808, *P* < 0.001), with those in Trial 1 having most orthodontic treatment.

**Table 3.** Distribution and significance of *t*-test of the mean dental index scores over surgical methods (Arms A, B, C, and D) within trials.

Eurocran index							
	Trial	Mean index score	*N*	Standard deviation	Range of mean score	*t*-test between Arms	*P*-value
Age 5 years	Trial 1	A 2.59	74	0.97	1.00–4.00	A vs B	0.14 ns
		B 2.36	68	0.91	1.00–4.00		
	Trial 2	A 2.47	69	0.98	1.00–4.00	A vs C	0.28 ns
		C 2.29	73	0.96	1.00–4.00		
	Trial 3	A 2.67	64	1.0	1.06–4.00	A vs D	0.90 ns
		D 2.65	70	0.91	1.00–4.00		
	All Trials 1, 2, 3	A, B, C, D 2.50	418	0.96	1.00–4.00		
Age 8 years	Trial 1	A 2.73	72	0.81	1.00–4.00	A vs B	0.146 ns
		B 2.53	73	0.87	1.00–4.00		
	Trial 2	A 2.48	67	0.94	1.00–4.00	A vs C	0.303 ns
		C 2.33	69	0.86	1.00–4.00		
	Trial 3	A 2.77	61	0.86	1.14–4.00	A vs D	0.897 ns
		D 2.75	69	0.81	1.00–4.00		
	All Trials 1, 2, 3	A, B, C, D 2.60	411	0.87	1.00–4.00		
Age 10 years	Trial 1	A 2.21	71	0.94	1.00–4.00	A vs B	0.21 ns
		B 2.02	71	0.92	1.00–4.00		
	Trial 2	A 2.30	68	1.01	1.08–4.00	A vs C	0.06 ns
		C 1.99	74	0.94	1.00–4.00		
	Trial 3	A 2.51	62	1.04	1.00–4.00	A vs D	0.53 ns
		D 2.62	64	0.98	1.08–4.00		
	All Trials 1, 2, 3	A, B, C, D 2.26	410	0.99	1.00–4.00		
5-year-olds' (5YO) index (at age 5 years) and GOSLON Yardstick scores (at age 8 and 10 years)							
	Trial	Mean index score	*N*	Standard deviation	Range of mean score	*t*-tests between Arms	*P*-value
Age 5 years*	Trial 1	A 2.86	74	0.94	1.06–4.88	A vs B	0.06 ns
		B 2.58	68	0.87	1.00–4.31		
	Trial 2	A 2.78	69	0.95	1.06–4.88	A vs C	0.11 ns
		C 2.52	73	0.94	1.00–4.94		
	Trial 3	A 2.94	64	1.04	1.31–5.00	A vs D	0.92 ns
		D 2.92	70	0.91	1.13–4.88		
	All Trials 1, 2, 3	A, B, C, D 2.77	418	0.95	1.00–5.00		
Age 8 years**	Trial 1	A 3.03	72	0.85	1.09–4.82	A vs B	0.137 ns
		B 2.82	73	0.81	1.36–4.82		
	Trial 2	A 2.78	67	0.9	1.18–4.91	A vs C	0.360 ns
		C 2.64	69	0.91	1.00–5.00		
	Trial 3	A 3.06	61	0.9	1.09–4.91	A vs D	0.850 ns
		D 3.08	69	0.86	1.64–5.00		
	All Trials 1, 2, 3	A, B, C, D 2.90	411	0.88	1.00–5.00		
Age 10 years	Trial 1	A 2.54	71	0.9	1.08–5.00	A vs B	0.18 ns
		B 2.34	71	0.88	1.00–4.92		
	Trial 2	A 2.49	68	1.02	1.17–4.50	A vs C	0.09 ns
		C 2.21	74	0.98	1.08–5.00		
	Trial 3	A 2.80	62	1.08	1.25–5.00	A vs D	0.45 ns
		D 2.94	64	1.03	1.50–5.00		
	All Trials 1, 2, 3	A, B, C, D 2.54	410	1.01	1.00–5.00		

*The data of the 5YO index is published previously in Heliövaara *et al.* (3).

**The data of the GOSLON Yardstick at age 8 years is published previously in Heliövaara *et al.* (4).

#### Comparisons between trials

Comparisons between trials detected significantly better mean index scores in Trial 2 Arm C (at all ages) and in Trial 1 Arm B (at 5 and 10 years of age) than in Trial 3 Arm D (Table 4). In addition, a significantly better 5YO index score was noted in Trial 1 Arm B than in Trial 3 Arm D.

**Table 4.** Significance of *t*-test of the mean dental index scores over surgical methods (Arms A, B, C, and D) between trials.

Eurocran index		*P*-value		*P*-value		*P*-value
Age 5 years	Arm A		Arm A		Arm A	
	Trial 1 vs Trial 2	0.464 ns	Trial 1 vs Trial 3	0.608 ns	Trial 2 vs Trial 3	0.234 ns
	Arm B vs Arm C		Arm B vs Arm D		Arm C vs Arm D	
	Trial 1 vs Trial 2	0.694 ns	Trial 1 vs Trial 3	0.055 ns	Trial 2 vs Trial 3	0.022 *
Age 8 years	Arm A		Arm A		Arm A	
	Trial 1 vs Trial 2	0.095 ns	Trial 1 vs Trial 3	0.802 ns	Trial 2 vs Trial 3	0.074 ns
	Arm B vs Arm C		Arm B vs Arm D		Arm C vs Arm D	
	Trial 1 vs Trial 2	0.159 ns	Trial 1 vs Trial 3	0.119 ns	Trial 2 vs Trial 3	0.003 **
Age 10 years	Arm A		Arm A		Arm A	
	Trial 1 vs Trial 2	0.582 ns	Trial 1 vs Trial 3	0.088 ns	Trial 2 vs Trial 3	0.259 ns
	Arm B vs Arm C		Arm B vs Arm D		Arm C vs Arm D	
	Trial 1 vs Trial 2	0.841 ns	Trial 1 vs Trial 3	0.000 ***	Trial 2 vs Trial 3	0.000 ***
5-Year-olds’ (5YO) index (at age 5 years) and GOSLON Yardstick scores (at age 8 and 10 years)						
		*P*-value		*P*-value		*P*-value
Age 5 years	Arm A		Arm A		Arm A	
	Trial 1 vs Trial 2	0.599 ns	Trial 1 vs Trial 3	0.652 ns	Trial 2 vs Trial 3	0.356 ns
	Arm B vs Arm C		Arm B vs Arm D		Arm C vs Arm D	
	Trial 1 vs Trial 2	0.732 ns	Trial 1 vs Trial 3	0.025 *	Trial 2 vs Trial 3	0.012 *
Age 8 years	Arm A		Arm A		Arm A	
	Trial 1 vs Trial 2	0.102 ns	Trial 1 vs Trial 3	0.851 ns	Trial 2 vs Trial 3	0.088 ns
	Arm B vs Arm C		Arm B vs Arm D		Arm C vs Arm D	
	Trial 1 vs Trial 2	0.214 ns	Trial 1 vs Trial 3	0.061 ns	Trial 2 vs Trial 3	0.004 **
Age 10 years	Arm A		Arm A		Arm A	
	Trial 1 vs Trial 2	0.757 ns	Trial 1 vs Trial 3	0.140 ns	Trial 2 vs Trial 3	0.097 ns
	Arm B vs Arm C		Arm B vs Arm D		Arm C vs Arm D	
	Trial 1 vs Trial 2	0.379 ns	Trial 1 vs Trial 3	0.000 ***	Trial 2 vs Trial 3	0.000 ***

*P < 0.05; ** *P* < 0.01; *** *P* < 0.001.

### Proportions of mean index score distributions

#### Dental arch indices

**Figure 4.**
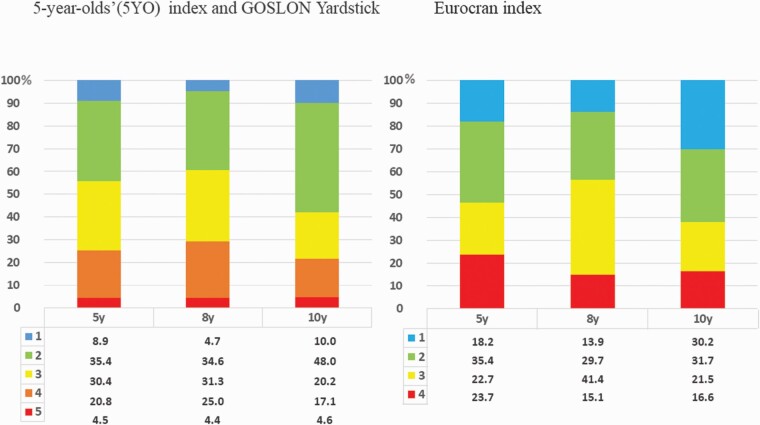
Proportions (%) of the mean dental index score distributions of the total sample at 5, 8, and 10 years of age.

Most of the models of the total sample (31.7–48%) were rated to category 2 with all indices at all ages, except for the Eurocran index at 8 years of age (Figure 4). At that age, 41.4% of the models were rated to Eurocran category 3. Overall, most of the models were rated to categories 2, 3, and 4 with all indices, except for the Eurocran index at 10 years of age. At 10 years of age, the second most common Eurocran index category (30.2%) was category 1.

A trend of a slightly more unfavourable distribution of index scores with increasing age from 5 to 8 years was noted. At the age of 10 years, the proportion of index scores 1 and 2 increased with both the Eurocran index and GOSLON Yardstick. The proportions of all patients at all ages in Eurocran and 5YO indices as well as in GOSLON Yardstick categories excellent and good (1, 2) varied between 39.3% and 61.9% and those in categories poor (4) between 15.1% and 25%. The proportion of patients in group 5, very poor (5YO index and GOSLON Yardstick), remained constant from 5 to 10 years (4.4–4.6%).

#### Eurocran palatal index

Most of the palatal scores (56.9%) were moderate (grade 2), 36.8% were good (grade 1), and 6.3% were poor (grade 3).

## Discussion

These are secondary reports of the dental arch relationships of the Scandcleft randomized trials in which three groups of centres tested four surgical protocols. This is the first follow-up study from 5 to 10 years of age and the first comparison of the dental indices used in the rating of the study models. The follow-up will continue until early adulthood.

### Primary surgery and orthodontic treatment

The results at 10 years of age agree with earlier findings of the Scandcleft study on dental arch relationships at the age of 5 and 8 years; none of the three trials revealed any significant differences within the trials between the common method and the alternative protocol regarding the dental arch relationship (3–6). However, significant differences were present between the trials at all ages. Comparisons between trials detected significantly better mean index scores in Trial 2 Arm C (at all ages) and in Trial 1 Arm B (at 5 and 10 years of age) than in Trial 3 Arm D.

When comparing the results of primary surgery on later dentofacial development, technique, staging, and sequencing of the palatal surgery are important. Other key variables that may affect surgical iatrogenesis are the skill and experience of the operating surgeon. A limitation of the Scandcleft study is the number and varying experience of the surgeons and the predominance of common method Arm A (2). There were 10 operating surgeons in Trial 3. On the other hand, most of the surgery in Trial 2 was performed by two senior high-volume plastic surgeons. When they used the method most familiar to them (Arm C), slightly more favourable results were achieved. It has been concluded that in experienced hands none of the four methods of closure of UCLP seem to bring any particular advantage or disadvantage compared with another method, and the familiarity and operator skill outweigh the importance of the surgical protocol (23). High-volume surgeons have been associated with more favourable outcomes than low-volume surgeons also earlier in a six-centre European international study (24) and in a national review of cleft care in the UK (25). In the latter, surgeons with 30 or more new referrals for primary surgery per year were compared with those who had less. The learning curve of a new surgical method should be considered especially when the case load is small (2).

Besides surgical iatrogenesis, genetics, congenital dysmorphology of the midface, other variations intrinsically associated with the cleft and functional adaptations may interfere with the normal growth pattern in individuals with clefts (26). An interesting recent finding with this same Scandcleft material is that agenesis of teeth at the age of 8 years has a significant impact on craniofacial growth and dental arch relationships (27). The number of individuals with GOSLON score 4–5 was 47.2% in the group with ≥2 missing maxillary teeth compared with those with no or only one missing maxillary tooth (26.1% and 26.3%, respectively). No significant difference was found for the presence or absence of the cleft lateral. The prevalence of missing cleft-side laterals at age 8 years was 43.8% (28). Another factor that evidently influenced the index scores of this study is orthodontic/orthopaedic treatment. At 10 years of age, 75.8% of the children had had orthodontic treatment. There was a significant difference in orthodontic treatment between the trials at age 10 years, with those in Trial 1 having most orthodontic treatment. Caution is needed when discussing the effect of the ongoing orthodontic treatment as factors such as type of appliances, length of treatment, co-operation, and experience of the orthodontist are not included. It has been suggested that all patients who have undergone orthodontic treatment should be excluded when presenting results with the GOSLON Yardstick (29). However, the purpose of this paper was also to compare the dental indices of the same group of patients at the same ages. The results did not differ between the indices.

Orthodontic treatment and correction of a crossbite may influence the indices positively. The greatest influence on the GOSLON score is from the anteroposterior assessment or overjet. The second and third determinants are vertical and transversal assessment, respectively. If there are dental compensations present such as proclination / retroclination of maxillary incisors or mandibular incisors, the score may shift to the next higher or lower score, depending on the magnitude of the compensation (20). In addition, orthodontic treatment with several different techniques and orthodontic appliances can make the scoring more difficult despite all raters of this study being cleft orthodontists. We plan to report more about the orthodontic treatment and the orthodontic results in the future.

### The indices

According to the World Health Organization, the ideal index should meet all of the following criteria: reliability, validity, and acceptance by the profession; lend itself to statistical analysis; and be administratively simple (30). In large multi-centre studies, intra-examiner weighted Kappa scores should be more than 0.8 and inter-examiner scores more than 0.7 to ensure that results are reliable (21). The Kappa scores of the dental indices were acceptable and comparable to those in previous inter-centre studies using the 5YO index and GOSLON Yardstick (3, 4, 10, 11, 17–20) and Eurocran index (12, 16). However, the reliabilities of the ratings varied individually although poor-quality models were excluded and the quality of the analysed dental models was good.

There are fewer studies using the Eurocran index than GOSLON Yardstick, which has been the most widely used index to assess dental arches since its introduction (31, 32). The reliability of the Eurocran was slightly better than that of the GOSLON Yardstick at 10 years of age, although the raters were less familiar with its use. However, the reliabilities of the palatal Eurocran index were questionable, and no further statistical analyses were performed. According to Jones *et al.* (21), the Eurocran palatal index showed no clear predictive validity.

Besides reliability, validity is essential for an index. Significant correlations between the Eurocran and 5YO indices and the Eurocran index and the GOSLON Yardstick existed at all ages. Correlations between the GOSLON Yardstick / 5YO index and the GOSLON Yardstick and MHB index have been shown earlier (33, 34). Evaluating the predictive validity of the indices is more challenging. In agreement with the earlier findings of the Scandcleft study (3–6), at 8 years of age the Eurocran index scores and their distribution were slightly less satisfactory than at 5 years of age. In 50 children with UCLP (35), the prevalence of anterior crossbite increased from 40% to 78%, and posterior crossbite from 66% to 76% in early mixed dentition irrespective of the arch configuration in the deciduous dentition. At 10 years of age the index scores were smallest and most of the patients had undergone orthodontic treatment.

At all ages, most of the children had index score 2, except for Eurocran index 3 at 8 years of age. The proportions of all patients at all ages in Eurocran and 5YO indices as well as in GOSLON Yardstick categories excellent and good (1, 2) varied between 39.3% and 61.9% and those in categories poor (4) between 15.1% and 25%. The proportion of patients in group 5, very poor (5YO index and GOSLON Yardstick), remained constant from 5 to 10 years (4.4–4.6%). These are patients that will most likely need orthognathic surgery later. It is to be expected that this proportion will be greater. The impairment of maxillary growth in UCLP continues into early adulthood. Only a small increase in length of the maxilla was observed between 5 and 18 years of age in a mixed longitudinal cephalometric study of 257 patients with complete UCLP (7). In the Eurocleft study, patients with UCLP with GOSLON scores of 3.5 and higher were considered candidates likely to require maxillary osteotomy at the completion of growth (19). In a retrospective longitudinal single-centre study with 70 patients with ULCP (36), the 5YO index score at 6 years of age was significantly worse in those with later orthognathic surgery (3.6 versus 2.4).

The predictive validity of the dental indices has been questioned. A systematic review of the predictive validity of the GOSLON Yardstick in UCLP (32) highlights the lack of consistent findings in the literature, the GOSLON Yardstick showing a predictive validity between 42.4% and 64.7%. The predictive validity was similar for MHB and 5YO indices and GOSLON Yardstick, with a 50–65% prediction of final outcome (at age 15–20 years) from 5 to 10 years (21). Recently, the reliability and predictive validity of the 5YO index and GOSLON Yardstick were evaluated in patients with UCLP at 5, 7/8, 10, 15/16, and 19 years (37). The predictive value of ‘good’ dental arch relationship scores (1 and 2) over time was good in all age groups (*n* = 106), whereas the prediction of cases in group 3 was very poor at all ages. Of the 5-year-olds allocated to group 3, 4, or 5, 60% had a good or fair dental arch relationship at 19 years. The use of the 5YO index before orthodontic treatment may help to predict outcome and clinical need for orthognathic surgery, especially in patients with the lowest and highest index scores (36).

### Strength and limitations

The strength of this randomized study is the large patient material of more than 400 Caucasian children with UCLP. The selection and allocation bias are reduced because of the randomization. Study limitations include the high number of operating surgeons, individual surgical learning curves and skill, lack of evaluation of the initial extent of cleft, agenesis of teeth and the amount of secondary surgery, and the type, length, and effect of orthodontic treatment. In addition, the age range of the patients at the time of the models, and the reliabilities of the individual ratings of the models varied. The final outcomes of dentofacial relations of the Scandcleft randomized trials can be evaluated after the growth is complete. Appraisal of other parameters, such as speech, facial appearance, patient satisfaction, and burden of care, should also then be included.

## Conclusions

The results of the three trials do not provide evidence that one surgical method is superior to the others when dental arch relationships are evaluated. The dental indices in this study could be used in evaluating, categorizing, and comparing the results of the surgical methods in children with UCLP. The results did not differ between the indices. The reliabilities of the dental indices were acceptable, and significant correlations existed between the dental indices at all ages. The reliability of the Eurocran palatal index was questionable.

## Funding

This study was supported by the Finnish Dental Society.

## Data Availability

The data underlying this article were provided by the cleft centres participating in the Scandcleft study. Data will be shared on request to the corresponding author with the permission of the centres.
